# P-133. Effect of Antibiotic Suppression for Prosthetic Valve Endocarditis on Recurrence Rates

**DOI:** 10.1093/ofid/ofaf695.360

**Published:** 2026-01-11

**Authors:** Catherine Cai, Michael Hovan, Catherine Ng, Zoe Verzani, Barry Hartman, Harjot K Singh

**Affiliations:** NewYork-Presbyterian Hospital/Weill Cornell Medical Center, New York City, NY; NewYork-Presbyterian Hospital/Weill Cornell Medical Center, New York City, NY; Weill Cornell Medical Center, New York City, New York; Weill Cornell Medical Center, New York City, New York; NewYork-Presbyterian Hospital/Weill Cornell Medicine, New York City, New York; Weill Cornell Medicine, new york city, New York

## Abstract

**Background:**

The prevalence of prosthetic valve endocarditis (PVE) has increased as more patients undergo valve replacement. Bacterial PVE can be difficult to treat because infected prosthetic material is often retained, but no formal guidelines exist regarding the use of suppressive antibiotics after the completion of initial treatment course. We sought to describe characteristics of patients with initial and recurrent PVE and the effects of antibiotic suppression on outcomes.

**Methods:**

We searched the electronic health record using ICD-10-CM diagnosis codes to identify patients with a new PVE diagnosis over an eight-year span at a large hospital in New York City. Patient charts were then manually reviewed to confirm the diagnosis of PVE and collect data of interest for statistical analysis, including demographic information, clinical characteristics, microbial data, and recurrences.

**Results:**

Among patients with PVE, a pathogen was identified in 174/184 (94.6%) cases. Only seven had a polymicrobial infection (7/184, 3.8%), yielding 191 isolates. The most common pathogens identified were Staphylococcus aureus (39/191, 20.4%), viridans Streptococci (36/191, 18.8%), Enterococcal species (33/191, 17.3%), and coagulase-negative Staphylococci (26/191, 13.6%).

Of 172 patients who completed a standard initial antibiotic treatment course, 69 (40.1%) were placed on antibiotic suppression afterward. The most common single agents used were amoxicillin (17/69, 24.6%), penicillin (15/69, 21.7%), dicloxacillin (9/69, 13%), and doxycycline (8/69, 11.6%). PVE recurrence was noted in 8/69 (11.6%) of patients on suppression, compared to 11/103 (10.7%) of patients who were not put on suppression, a difference that was not statistically significant.

**Conclusion:**

Patients who are already higher risk for recurrence due to age, comorbidities, or other factors may be more likely to be placed on suppression, which potentially confounds our findings. Additional analyses will investigate if suppression correlates with differences in rehospitalization and mortality rates, as well as whether suppression is associated with adverse events such as increased rates of *Clostridioides difficile* infection.

**Disclosures:**

All Authors: No reported disclosures

